# Transition from Depression to Suicidal Attempt in Young Adults: The Mediation Effect of Self-Esteem and Interpersonal Needs

**DOI:** 10.3390/ijerph192114342

**Published:** 2022-11-02

**Authors:** Xingyun Liu, Miao Liu, He Li, Liuling Mo, Xiaoqian Liu

**Affiliations:** 1 Key Laboratory of Adolescent Cyberpsychology and Behavior, Ministry of Education, Key Laboratory of Human Development and Mental Health of Hubei Province, School of Psychology, Central China Normal University, Wuhan 430079, China; 2 CAS Key Laboratory of Behavioral Science, Institute of Psychology, Chinese Academy of Sciences, Beijing 100101, China

**Keywords:** depression, suicidal attempt, self-esteem, thwarted belongingness, perceived burdensomeness

## Abstract

Background: Depression increases the risk of suicide. Depression and suicide attempts are significantly impacted by low self-esteem and interpersonal needs (i.e., thwarted belongingness (TB) and perceived burdensomeness (PB)). More research is required to clarify how these factors affected the change from depression to suicidal attempts, which would dramatically lower the suicide fatality rate. We sought to examine the mediating roles of self-esteem, TB, and PB in Chinese young adults, since previous research shows that self-esteem has a strong relationship with TB, while TB and PB have strong relationships with suicide attempts. Methods: Measures on depression, interpersonal needs, and attempted suicide were completed by a sample of 247 Chinese social media users who had stated suicidal ideation online. Results: The findings showed that people who attempted suicide had significantly higher levels of TB and PB. Suicidal attempts were also impacted by depression via the mediational chains, which included self-esteem, TB, and PB. Conclusions: Our findings might contribute to the expansion of the interpersonal theory of suicide and have an impact on effective suicide prevention.

## 1. Introduction

Approximately 700,000 people die of suicide every year [1]. In contrast to many other causes of death, for which higher-resourced settings are in relatively favorable condition, suicide mortality has resulted in considerable life losses but has seen little progress in many higher-resourced settings since 2000 [1]. Young people are likewise in a critical condition. Male suicide rates rose with age, and female suicide rates rose with age starting at 30 but peaked among those between the ages of 15 and 29 [2]. Among people under the age of 29, suicide is the leading cause of death [3]. Each year, 200 million people attempt suicide in China, two-thirds of them between the ages of 15 and 34 [4]. Prevention of suicide is absolutely essential, especially for young people.

People who have tried suicide in the past have a 38 to 100 times higher risk of doing so again than the general population [5,6,7]. Researchers also concur that depression and suicidal behaviors are closely associated [8]. Despite the lack of consensus in this area, some investigations discovered that the only trajectory that can predict suicide attempts with the highest mean scores and greatest temporal variability is the depressive symptom trajectory [9]. Depressive people have a suicide rate that is around 20 times greater than the overall population [10]. Despite the fact that there is a lot of research showing a connection between depression and suicide, most people who experience depression do not kill themselves. While common risk indicators for suicide behavior have been identified, efforts to accurately forecast which people would engage in or pass away from suicidal behaviors are woefully insufficient [11]. The focus of current research in this area is on the co-occurring disorders of depressed suicide attempters [12,13], the genetic influences on depression and suicidal attempts [14], the impact of traumatic life events [15,16,17], and so forth. However, it is still unclear how depression and suicide attempts are related. In order to increase accurate suicide risk assessment and prevention, it is essential to understand the mechanism that indicates how depression proceeds into a suicidal attempt (not suicidal ideation or general suicide risk).

According to the interpersonal theory of suicide (ITS), (1) stressful life events, mental illnesses, and other risk factors for suicide are relatively more distal in the causal chain of risk factors for suicide than thwarted belongingness (TB) and perceived burdensomeness (PB), which are proposed to be the most proximal mental states that precede the development of suicidal ideation. (2) Self-esteem influences suicide behaviors through PB as a component of self-hatred. (3) Suicidal ideation is explained by TB and PB, two separate but connected variables, whereas suicide attempts are caused by suicide capabilities. (4) Suicide attempts can influence one’s ability to commit suicide [18].

Despite the abundance of research demonstrating a connection between low self-esteem and loneliness [19,20], loneliness is an element of TB according to the ITS, low self-esteem is classified as PB, and the ITS has not provided an explanation for the connection between PB and TB [18]. As far as we know, no initiatives have been taken to study the mechanism. What are the consequences of TB and PB on suicidal capabilities, such as suicidal attempts, aside from fear of death and physical pain tolerance described by the ITS, which can enhance suicidal capabilities? Previous studies revealed that loneliness (cf., TB) and self-hate (cf., PB) are both major determinants of suicidal attempts [21,22,23,24]. More research is necessary to comprehend the aforementioned problems in their entirety.

Individuals suffering from depression are at a higher risk of suicidal attempts [9]. A hallmark feature of depression is the loss of confidence or self-esteem [25]. Moreover, according to the ITS, TB and PB are the most proximal factors that precede the development of suicidal behaviors, while self-esteem is relatively more distal. That is, for the depressed, depression may predict suicidal attempts through the mediating chains of self-esteem and interpersonal needs (i.e., TB and PB). To be specific, self-esteem refers to “the individual’s positive or negative attitude toward the self as a totality” [26]. Existing research has demonstrated that individuals with low self-esteem have little confidence in their ability to begin and maintain satisfactory reciprocal care in social settings, i.e., self-esteem has a close association with social disengagement [27], which is a symptom of TB. The Japanese Hikikomori Phenomenon has gained increasing attention in recent years. The Hikikomori Phenomenon refers to young people who disengage socially for an extended period of time. China has already been affected by this problem [28]. Young people’s social withdrawal has caused an elevated rate of unemployment [29], which has been categorized into PB by the ITS [18]. As a result, we inferred that TB may act as a mediator between self-esteem and PB.

One of the best indicators of suicide death is a history of attempted suicide [18]. Why does the current research abovementioned prefer to concentrate on overall suicide risk rather than more precise markers such as suicidal attempts? The suicide attempt analysis may be challenging due to the extremely large sample sizes needed compared to the extremely low base rates of suicide attempts and fatalities in the general population [30], not to mention the difficulty in locating enough depressed individuals with a history of suicide attempts. An innovative answer to this issue is provided by machine learning algorithms and the Internet. To proactively identify suicidal social media users and provide confidential crisis management to them, we devised a novel method called the Proactive Suicide Prevention Online (PSPO). The PSPO has been evaluated as efficient and beneficial in identifying suicidal people and providing crisis intervention [31]. With the assistance of the PSPO, we can find young people with a history of suicidal attempts timely and easily.

In conclusion, because it is unclear and possibly contradictory in this area, this paper aims to investigate the transition from depression to suicide attempts and the mediating effect of self-esteem, TB, and PB among them. Additionally, more research is needed to investigate the applicability of ITS for young Chinese with suicidal risk. We concentrated on self-esteem, TB, and PB in this paper in an effort to shed light on the transmitting path based on ITS. In an online sample of Chinese people, we formulated the following hypotheses: depression would be a predictor of suicidal attempts through the mediating chains of self-esteem, TB, and PB.

## 2. Materials and Methods

### 2.1. Participants

By including the link to the surveys in direct messages, we were able to use PSPO to gather data to evaluate the aforementioned hypothesis. A total of 12,486 social media users who were recognized as publishing suicidal posts were sent direct messages with the questionnaire link between 3 July 2017 and 3 July 2018. A total of 4318 (34.58%) people completed the evaluation protocol without receiving any payment after giving their informed consent. A sum of 260 (6.02%) of them simultaneously filled out all the questionnaires for this study after removing replies that indicated incomplete information (*n* = 1, 0.38%) and the absence of depression as reported by the participants (*n* = 12, 4.62%) (i.e., they received a total score less than 13 for the Beck Depression Inventory-II or 5 for the Patient Health Questionnaire-9); there were 247 valid samples remaining with various levels of depression. Ethical approval was obtained from the Institute of Psychology, Chinese Academy of Sciences, and Central China Normal University.

### 2.2. Measures

Participants’ demographic information, which included gender, age, education level, marital status, and employment status, was obtained.

Depression was measured by the 21-item version of the Beck Depression Inventory-second edition (BDI-II) and the Patient Health Questionnaire-9 (PHQ-9). For BDI-II, each item is rated on a 4-point scale from 0 to 3, with a total score ranging from 0 to 63. Each item depicts a particular behavioral expression of depression. People were classified as not depressed from 0 to 13, mildly depressed from 14 to 19, moderately depressed from 20 to 28, and seriously depressed from 29 to 63. Sample item includes, “0 I do not feel sad. 1 I feel blue or sad. 2 I am blue or sad all the time and I can’t snap out of it. 3 I am so sad or unhappy that I can’t stand it”. Participants need to select one of the four options that best describes their circumstances for the previous week. The BDI-II’s original and Chinese versions both displayed high levels of validity and reliability [32,33]. This study had a strong internal consistency (α = 0.85).

For PHQ-9, “Having little interest or pleasure in doing things” is an example item. Participants rated the frequency of the nine symptoms during the past two weeks on a 4-point Likert scale. The overall PHQ-9 score ranges from 0 to 27, with higher scores indicating more severe depressive symptoms. People who scored 0 to 4 are classified as not depressed, 5 to 9 as mildly depressed, 10 to 14 as moderately depressed, 15 to 19 as moderately severe depressed, and 20 to 27 as significantly depressed. The Chinese version has been shown to have good psychometric properties [34], and the internal consistency of this study was 0.80. Since some researchers have proposed that certain BDI-II items (i.e., items 5, 6, 7, and 8) are utilized to measure self-esteem, it is incorrect to use the BDI-II total score to evaluate self-esteem directly [32,35]. As a result, we conducted our study using the PHQ-9 mean score. 

Suicidal attempt was measured by one item: “Have you ever tried to kill yourself?”. Participants gave binary responses (yes/no). Participants were classified as belonging to the suicide attempt group if they had attempted suicide; otherwise, they were placed in the only depressive group.

Self-esteem was measured by the 10-item version of the Rosenberg Self-Esteem Scale (RSES) [36]. To gauge the level of self-esteem, each item is scored on a 4-point scale ranging from 1 (strongly disagree) to 4 (strongly agree). “I feel that I am a person of worth, at least on an equal plane with others.”, and “I feel that I have a number of good qualities.” are sample items. Higher self-esteem is indicated by a higher score. The RSES’s original and Chinese versions both displayed good validity and reliability [37,38]. The internal consistency in this study was high (α = 0.84).

Interpersonal needs were measured by the 15-item version of the Interpersonal Needs Questionnaire (INQ) (17). Based on a 7-point Likert scale (1–7), a total of 9 items measure TB, for example, “These days I think I am a burden on society”, and 6 items measure PB, for example, “These days I think the people in my life wish they could be rid of me”. The INQ’s original and Chinese version both displayed high reliability and validity [39,40]. In this study, the internal consistency values for the total scale and two subscales were 0.85, 0.79 and 0.88, respectively.

### 2.3. Data Analysis

The statistical analysis was conducted using Mplus7 and SPSS21. Descriptive statistics for demographic characteristics and study variables were also tabulated. Bivariate correlations were conducted. To evaluate the mediation model, path analysis (PA) was used. Model fit was evaluated using several fit indices: (1) the chi-square (χ^2^) test of model fit, (2) the root mean square error of approximation (RMSEA), (3) the comparative fit index (CFI), (4) the Tucker–Lewis index (TLI), and (5) weighted root mean square (WRMR), due to the presence of binary data. An RMSEA value of less than 0.06, CFI at or above 0.90, TLI at 0.90 or higher, and WRMR value that was below 1.00 indicate a relatively good fit [41,42]. The Monte Carlo method was used to analyze the mediation effect, which can lessen the problem of high dimensionality in large systems by three steps: (1) power system random state generation, (2) power deficit minimization, and (3) reliability indices calculation [43].

## 3. Results

The demographic characteristics of participants are displayed in Table 1. As shown, there was no difference between the two groups in demographic variables. The majority of participants were young, single, female students.

The range of the BDI-II’s total score was 15 to 60 (M = 35.66, SD = 9.60). A total of 13 participants were classified as mildly depressed, 50 participants fell into the moderate depression category, and 184 participants had severe depression. The range of the PHQ-9′s total score was 5 to 27 (M = 18.47, SD = 5.43). Four categories of depression were identified as mentioned above: mild depression (14 participants), moderate depression (48 participants), moderately severe depression (67 participants), and severe depression (118 participants). The correlation between BDI-II and PHQ-9 was 0.68 (*p* < 0.001).

The group differences in study variables by suicide status are shown in Table 2. The suicide attempt group reported higher levels of TB (*t* = −3.12, *p* = 0.002) and PB (*t* = −4.13, *p* < 0.001) than the only depressive group, while there was no significant difference between the two groups on depression (*t* = −1.34, *p* = 0.18) and self-esteem (*t* = 1.38, *p* = 0.17).

Descriptive statistics and correlations of study variables are displayed in Table 3. As shown, depression was significantly correlated with self-esteem (*r* = −0.42, *p* < 0.001). The two factors of interpersonal needs were significantly correlated with suicidal attempt (i.e., TB (*r* = 0.20, *p* = 0.002) and PB (*r* = 0.26, *p* < 0.001)) and self-esteem (i.e., TB (*r* = −0.33, *p* < 0.001) and PB (*r* = −0.48, *p* < 0.001)). Therefore, we conducted subsequent mediation analysis with path analysis.

**Figure 1:**
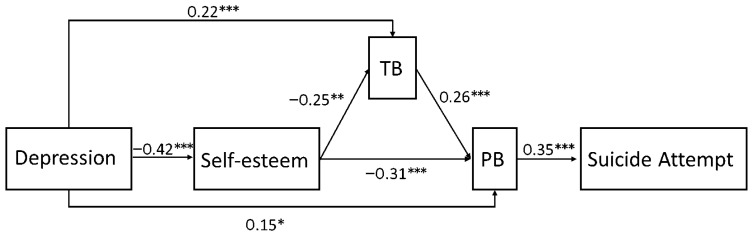
The mediating effect of self-esteem and interpersonal needs in the association between depression and suicidal attempt. * *p* < 0.05, ** *p* < 0.01, *** *p* < 0.001.

Results of PA are depicted in Figure 1. Model indexes were acceptable, with $\chi^{2}$/df = 1.34, RMSEA = 0.04, CFI = 0.99, TLI = 0.98, and WRMR = 0.43. Depression was associated with a lower level of self-esteem (β = −0.42, *p* < 0.001) and a higher level of interpersonal needs (TB: β = 0.22, *p* < 0.001; PB: β = 0.15, *p* = 0.01). Self-esteem was associated a lower level of interpersonal needs (TB: β = −0.25, *p* < 0.001; PB: β = −0.31, *p* < 0.001). Meanwhile, only PB was significantly associated with suicidal attempt (β = 0.35, *p* < 0.001). The relation between self-esteem and PB was mediated by TB. In summary, these associations supported the hypothesis that depression predicted suicidal attempt through the mediating chains of self-esteem, TB, and PB.

Further mediation analysis with the Monte Carlo methods found significant indirect effects of (1) self-esteem, TB, and PB as the mediators between depression and suicidal attempt, (2) self-esteem and PB as the mediators between depression and suicidal attempt, (3) PB as the mediator between depression and suicidal attempt, (4) self-esteem and TB as the mediator between depression and PB, and (5) self-esteem as the mediator between depression and PB, (6) self-esteem as the mediator between depression and TB. The estimated mediation effects are shown in Table 4, which provide support for the hypothesis.

**Figure 2:**
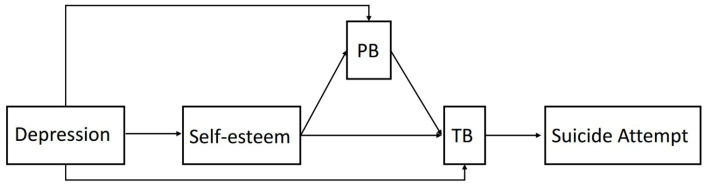
The alternative model that self-esteem predicts TB through the mediation of PB.

**Figure 3:**
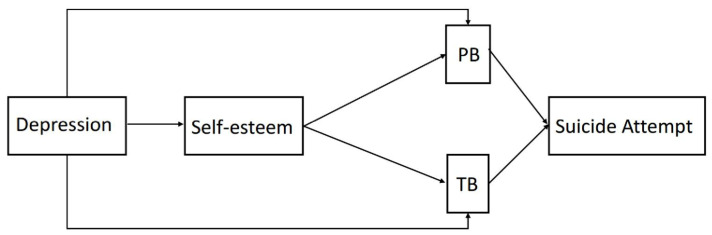
The alternative model that self-esteem predicts TB and PB parallelly.

**Figure 4:**
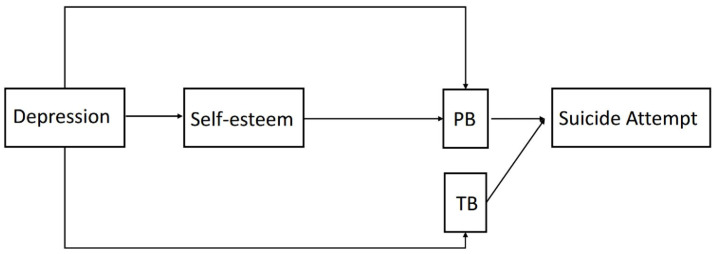
The alternative model that self-esteem just predicts PB according to ITS.

To further verify the model in Figure 1, we also tested the following three models: (1) self-esteem predicted TB through the mediation of PB (Figure 2), and (2) self-esteem predicted TB and PB parallelly (Figure 3). (3) Self-esteem just predicted PB according to the ITS (Figure 4). Neither of the three alternative models reached the model fit indices with $\chi^{2}$/df = 2.62, RMSEA = 0.08, CFI = 0.97, TLI = 0.90, and WRMR = 0.62 for Figure 2, $\chi^{2}$/df = 11.63, RMSEA = 0.23, CFI = 0.93, TLI = 0.25, and WRMR = 0.68 for Figure 3, and $\chi^{2}$/df = 15.02, RMSEA = 0.24, CFI = 0.83, TLI = 0.17, and WRMR = 1.38 for Figure 4. Those results further demonstrate the feasibility of the model in Figure 1.

## 4. Discussion

This study aimed to testify the ITS in Chinese young people that could distinguish the only depressive group from the suicide attempt group and illuminate the transition mechanisms that led from depression to suicidal attempts. We found that in contrast to the only depressive group, the suicide attempt group reported a higher level of TB and PB. More importantly, the effect of depression on suicidal attempt was mediated by self-esteem, TB, and PB. Our findings make a theoretical contribution to the field by analyzing TB and PB’s effect on suicidal capability and the relationship between self-esteem and PB in the Chinese depressive social media user population.

Consistent with previous research [44], no difference in gender, age, education level, marital status, or working status between the only depressive group and the suicide attempt group among Chinese social media users was found. Our findings also indicated that demographic characteristics are not helpful in distinguishing the only depressive group from the suicide attempt group in Chinese social media users, and further markers would be required to differentiate these two groups [45]. As expected, the suicide attempt group demonstrated a higher level of TB and PB than the only depressive group, which is consistent with previous studies [46,47]. Interestingly, the level of depression and self-esteem had no significate difference. One possible reason may be due to that all participants were suicidal and depressed after screening. Depression and self-esteem have a close reciprocal relation, and low self-esteem is one of the features of depression [25], insofar as both groups’ score for self-esteem was lower than 2 (M = 1.93 for the depression, M = 1.84 for the attempted) in our study. This phenomenon also suggests that unlike TB and PB, depression and self-esteem do not play a role in screening for suicidal attempts in depressive groups. Because the mechanism among Chinese young people that contributes to the transition from depression to suicidal attempts is not crystal clear, more research is required to investigate how these two groups are different in terms of self-esteem, TB, PB, and other factors (e.g., biology, personality traits, and so on). Our findings suggest that the identification of high-risk cases will be facilitated by the TB and PB of depressed individuals.

Interestingly, we found that depression could predict suicidal attempts through the mediating chains of self-esteem, TB, and PB. Furthermore, our study demonstrated that the associations of depression with PB were mediated by self-esteem and TB, even when compared to alternative models. Moreover, it is also worth noting that the mediation between depression and TB through self-esteem and the mediation between depression and PB through self-esteem were both significant. Those results were different from the ITS. Our mediation findings also shed light on how suicide capacities, i.e., suicide attempts, are associated with TB and PB, potentially complementing the ITS. This theory states that suicide capability is acquired through habituation to fear and pain involved in death [18]. However, the real world is a lot more complicated. Depressive people have low self-esteem, and with the co-occurrence TB and PB, they would have fewer reasons to live and more reasons to attempt suicide. Low levels of self-esteem, TB, and PB may accumulate courage for suicidal people to take action to kill themselves. Our study expands ITS to a depressive Chinese young adult sample with suicidal risk by providing empirical evidence to confirm that self-esteem, TB, and PB are alarm signals that indicate the depressive people’s intent to commit suicide. As a result, one potential clinical implication of these findings is to pay closer attention to depressive individuals with high levels of TB and PB because they may have a higher risk of suicide. At the same time, the findings of the study may supplement current gatekeeper training. It might be applicable, for instance, to a campus environment. If it is feasible, teachers, practitioners, and administrators may not only utilize social media posts as indications to keep an eye on their students’ health. They could also educate them to show solicitude for their schoolmates who were depressed in school and posted online about being isolation and burdensome as well as how to properly report such situations to professionals. While the study has made several contributions to the existing research, there still exist several limitations. Firstly, participants in this study were mainly college-educated, young, single women who were depressive. One possible reason is that according to the WHO, suicide attempts are about two to four times more frequent among females than males [2]. Further analyses would be required to generalize the findings to other populations of social media users with different demographic characteristics and suicidal situations. Moreover, while the relation between self-esteem and PB is established in numerous countries [19,20], further research is required to confirm these findings in various cultural contexts. Secondly, we only used PHQ-9 in our analysis, but BDI-II could be analyzed in the future study. For example, an earlier study demonstrated that anhedonia measured by the BDI-II has a close relationship with suicidal risk [48]. Meanwhile, more items could be utilized in the future to boost the measurement’s validity, as we only used one item to measure suicide attempts in our study. Thirdly, while 247 is a sizable sample, it is still not enough for the Structural Equation Modelling (SEM) analysis. Future studies should consider using SEM to decrease measurement error [49]. Fourthly, the relationships shown in this study are correlational in nature due to the cross-sectional research approach to investigate psychiatric symptoms. The impact of self-esteem, TB, and PB discovered in this study would need to be investigated further using longitudinal designs [50] and case control studies. Finally, this study relied on self-reported data, which is susceptible to bias. Future studies may take ethics, practical application, and legal duty into account while using more unbiased indicators [51].

## 5. Conclusions

This study aimed to investigate the interaction between self-esteem, TB, and PB for distinguishing the only depressive group from the suicide attempt group and elucidate the mechanism that transits from only depressive to the suicide attempt. Our findings demonstrated that suicide attempters were found to be with a significantly higher level of TB and PB. Moreover, self-esteem TB and PB are chain mediators that convert depression into suicidal attempts. TB mediates the relation between self-esteem and PB. These findings broaden ITS by elucidating the connections between PB and self-esteem via the mediating function of TB. This work provides a way for future research to concentrate on the theoretical aspects in the identification of suicidal attempters, ultimately assisting in the establishment of accurate and effective suicide prevention.

## Figures and Tables

**Table 1:** Demographic characteristics of participants by suicidal status.

	Total (*N* = 247) *n* (%)/M ± SD	Only Depressive (*n* = 156) *n* (%)/M ± SD	Suicide Attempt (*n* = 91) *n* (%)/M ± SD	$\chi^{2}/t$	*p*
Gender				0.42	0.52
Male	36 (14.57%)	21 (13.46%)	15 (16.48%)		
Female	211 (85.43%)	135 (86.54%)	76 (83.52%)		
Age (years)	20.89 ± 4.66	20.81 ± 4.21	21.02 ± 5.41	−0.34	0.73
Education level				5.89	0.053
Primary	23 (9.31%)	11 (7.05%)	12 (13.19%)		
Secondary	129 (52.23%)	77 (49.36%)	52 (57.14%)		
Tertiary	95 (38.46%)	68 (43.59%)	27(29.67%)		
Marital status				2.88	0.09
Single	196 (79.35%)	129 (82.69%)	67 (73.63%)		
Not single	51 (20.65%)	27 (17.31%)	24 (26.37%)		
Working status				3.32	0.19
Working	67 (27.13%)	44 (28.21%)	23 (25.27%)		
Not working	55 (22.27%)	29 (18.59%)	26 (28.57%)		
Studying	125 (50.61%)	83 (53.21%)	42 (46.15%)		

**Table 2:** Group differences on study variables by suicidal status.

	Only Depressive (*n* = 156) M ± SD	Suicide Attempt (*n* = 91) M ± SD	*t*	Effect Size
Depression	2.01 ± 0.60	2.12 ± 0.61	−1.34	0.18
Self-esteem	1.93± 0.47	1.84 ± 0.44	1.38	0.20
TB	4.96 ± 1.06	5.40 ± 1.08	−3.12 **	0.41
PB	4.47 ± 1.58	5.30 ± 1.40	−4.13 ***	0.56

TB = thwarted belongingness, PB = perceived burdensomeness, ** *p* < 0.01, *** *p* < 0.001.

**Table 3:** Descriptive statistics and bivariate correlations of the study variables.

		M ± SD *n* (%)	1	2	3	4
1.	Depression	2.05 ± 0.60	—			
2.	Suicidal Attempt	91 (36.84%)	0.09	—		
3.	Self-esteem	1.89 ± 0.46	−0.42 ***	−0.09	—	
4.	TB	5.12 ± 1.09	0.32 ***	0.20 **	−0.33 ***	—
5.	PB	4.78 ± 1.57	0.38 ***	0.26 ***	−0.48 ***	0.39 ***

TB = thwarted belongingness, PB = perceived burdensomeness, *** *p* < 0.001, ** *p* < 0.01.

**Table 4:** Estimated mediation effects of self-esteem and interpersonal needs in the association between depression and suicidal attempt.

Mediation Effect	Indirect Effect	95% Confidence Interval
Depression → Self-esteem → TB → PB → Suicidal Attempt	0.01 *	[0.002, 0.03]
Depression → Self-esteem → PB → Suicidal Attempt	0.04 **	[0.03, 0.12]
Depression → PB → Suicidal Attempt	0.05 *	[0.01, 0.16]
Depression → Self-esteem → TB → PB	0.03 **	[0.02, 0.12]
Depression → Self-esteem → PB	0.13 ***	[0.18, 0.47]
Depression → Self-esteem → TB	0.10 **	[0.08, 0.30]

TB = thwarted belongingness, PB = perceived burdensomeness, * *p* < 0.05, ** *p* < 0.01, *** *p* < 0.001.

## Data Availability

The data presented in this study are available on request from the corresponding author. These data are not publicly available in the interest of ethics.
